# High Exploratory Phenotype Rats Exposed to Environmental Stressors Present Memory Deficits Accompanied by Immune-Inflammatory/Oxidative Alterations: Relevance to the Relationship Between Temperament and Mood Disorders

**DOI:** 10.3389/fpsyt.2019.00547

**Published:** 2019-08-05

**Authors:** Camila Nayane de Carvalho Lima, Francisco Eliclécio Rodrigues da Silva, Adriano José Maia Chaves Filho, Ana Isabelle de Gois Queiroz, Adriana Mary Nunes Costa Okamura, Gabriel Rodrigo Fries, João Quevedo, Francisca Cléa F de Sousa, Silvania Maria Mendes Vasconcelos, David F de Lucena, Marta Maria de França Fonteles, Danielle S. Macedo

**Affiliations:** ^1^Neuropharmacology Laboratory, Drug Research and Development Center, Department of Physiology and Pharmacology, Faculty of Medicine, Universidade Federal do Ceara, Fortaleza, Brazil; ^2^Translational Psychiatry Program, Department of Psychiatry and Behavioral Sciences, McGovern Medical School, The University of Texas Health Science Center at Houston (UTHealth), Houston, TX, United States; ^3^Department of Pharmacy, Faculty of Farmacy, Dentistry and Nursing, Universidade Federal do Ceara, Fortaleza, Brazil; ^4^National Institute for Translational Medicine (INCT-TM, CNPq), Neurosciences and Behavior Department, Faculdade de Medicina de Ribeirão Preto (FMRP), Ribeirão Preto, Brazil

**Keywords:** temperament, animal model, bipolar disorder, paradoxical sleep deprivation, unpredictable stress, neuroinflammation, memory impairment

## Abstract

Low-exploratory (LE) and high-exploratory (HE) rodents mimic human depressive and hyperthymic temperaments, respectively. Mood disorders (MD) may be developed by the exposure of these temperaments to environmental stress (ES). Psychiatric symptoms severity in MD patients is related to the magnitude of memory impairment. Thus, we aimed at studying the consequences of the exposure of LE and HE male Wistar rats, during periadolescence, to a combination of ES, namely, paradoxical sleep deprivation (PSD) and unpredictable stress (US), on anxiety-related behavior in the plus maze test, working (WM) and declarative memory (DM) performance. We also evaluated hippocampal immune-inflammatory/oxidative, as consequences of ES, and prevention of ES-induced alterations by the mood-stabilizing drugs, lithium and valproate. Medium exploratory (ME) control rats were used for comparisons with HE- and LE-control rats. We observed that HE-controls presented increased anxiolytic behavior that was significantly increased by ES exposure, whereas LE-controls presented increased anxiety-like behavior relative to ME-controls. Lithium and valproate prevented anxiolytic alterations in HE+ES rats. HE+ES- and LE+ES-rats presented WM and DM deficits. Valproate and lithium prevented WM deficits in LE-PSD+US rats. Lithium prevented DM impairment in HE+ES-rats. Hippocampal levels of reduced glutathione (GSH) increased four-fold in HE+ES-rats, being prevented by valproate and lithium. All groups of LE+ES-rats presented increased levels of GSH in relation to controls. Increments in lipid peroxidation in LE+ES- and HE+ES-rats were prevented by valproate in HE+ES-rats and by both drugs in LE+ES-rats. Nitrite levels were increased in HE+ES- and LE+ES-rats (five-fold increase), which was prevented by both drugs in LE+ES-rats. HE+ES-rats presented a two-fold increase in the inducible nitric oxide synthase (iNOS) expression that was prevented by lithium. HE+ES-rats showed increased hippocampal and plasma levels of interleukin (IL)-1β and IL-4. Indoleamine 2, 3-dioxygenase 1 (IDO1) was increased in HE+ES- and LE+ES-rats, while tryptophan 2,3-dioxygenase (TDO2) was increased only in HE+ES-rats. Altogether, our results showed that LE- and HE-rats exposed to ES present distinct anxiety-related behavior and similar memory deficits. Furthermore, HE+ES-rats presented more brain and plasma inflammatory alterations that were partially prevented by the mood-stabilizing drugs. These alterations in HE+ES-rats may possibly be related to the development of mood symptoms.

## Introduction

The relationship between personality and mood disorders has been studied for decades. Indeed, Emil Kraepelin described four basic affective dispositions, namely, depressive, manic (hyperthymic), cyclothymic, and irritable, and proposed that imbalances between these affective temperaments could be the cause of mental disorders (1). Several decades later, the personality model proposed by Cloninger (2) and its derived instruments have been widely applied to mood disorders (3, 4). According to Cloninger’s model, temperament is the emotional core of personality and represents the basic pattern of response to emotional stimuli that is heritable and moderately stable through life. Based on Cloninger classification, temperament is divided into four dimensions, namely, novelty seeking (NS), harm avoidance (HA), reward dependence (RD), and persistence (P) (5). NS and HA are dimensions evolutively conserved in humans and mammals and greatly influence exploratory behavior. This behavior consists of a complex act that allows the collection of information about the environment and increases the chances to find food, mating partner, shelter, and, lately, of survival (6, 7).

In preclinical research, the selection based on exploratory behavior has been applied as a useful approach to study temperament (8). This selection allows the separation of rodents in two extreme subgroups—high and low exploratory (named here HE and LE, respectively). This trait is stable over time and keeps important associations with other anxious and depression-like behaviors as well as response to psychoactive drugs (8–10). In humans, several studies reported that patients diagnosed with mood disorders (both unipolar depression and bipolar disorder) present high HA, while bipolar patients differentially express high NS and RD domains (11, 12). In this context, the selection of extreme temperamental features (based on exploratory behavior) has emerged as a valuable tool to study the biological basis of temperament and to understand the involvement of personality traits in adaptive responses to stress and stress-related disorders.

Despite the well-documented consequences of paradoxical sleep deprivation (PSD) in animal models, the effects of combining environmental contingencies in a model induced by sleep deprivation associated with stress in memory and learning have been neglected. Sleep deprivation associated with unpredictable stressors occurs more commonly in the modern society, thus representing a better translational model.

Previous reports show that sleep deprivation induces increases in brain oxidative stress. For instance, authors reported that 72 h of rapid eye movement (REM) sleep deprivation increases lipid oxidation in both hippocampal and cortex areas (13). In another study, 21 days of intermittent REM sleep was shown to increase lipid oxidation and decrease superoxide dismutase (SOD) and glutathione peroxidase (GPx) activity in rat hippocampus (14). In contrast, Ramanathan et al. (15) showed that sleep deprivation increases antioxidants in the hippocampus, cerebellum, and neocortex.

Additionally, it is well known that allosteric loading conditions to the central nervous system (CNS), such as sleeping deprivation and unpredictable stress, may lead to activation of microglial activity and thereby aid release of a variety of proinflammatory and neurotoxic factors, including cytokines, such as tumor necrosis factor (TNF-α), interleukin (IL) -1β, IL-6, and free radicals, such as nitric oxide (NO) and superoxide, resulting in a decline in cognitive function (16–20).

These proinflammatory cytokines lead to an increased NO production *via* direct activation of microglial inducible nitric oxide synthase (iNOS) and indirect activation of neuronal NOS (nNOS) through N-methyl-D-aspartate (NMDA) receptor complex activation (NMDA2R subunit) (21, 22). Furthermore, an increased release of proinflammatory cytokines, like IL-1β, has been shown to bring about an increase in quinolinic acid to kynurenic acid ratio, as well as an inhibition of excitatory amino acid (glutamate) removal *via* focal loss of astroglial EAAT1/2 (excitatory amino acid transporter), leading to a net NMDA agonism (21, 23).

Lithium (Li) and valproic acid (VPA) are classical mood-stabilizing agents that are effective as maintenance therapy in mood disorders. Consistent evidence has shown that these drugs present neuroprotective action against several neuropathology models, which seems mainly due to their well-demonstrated antioxidant, anti-inflammatory, and neurotrophic properties (24–26). Regarding stress models, Li and VPA individually rescued the effects of chronic unpredictable stress on behavior and brain oxidative homeostasis (27, 28). Also, Li and VPA prevented the oxidative damage, mitochondrial dysfunction, and hypothalamic–pituitary–adrenal (HPA) hyperactivity induced by sleep deprivation (29, 30). Additionally, both these mood stabilizers protected against sleep deprivation-induced mania-like phenotype (29), while Li rescued some sleep deprivation-associated cognitive deficits (31). However, little is known about the effects of these mood-stabilizing drugs on behavioral and neurochemical consequences of the combination of sleep deprivation and unpredictable stress as well as the influence of temperament in this effect.

In the present study, we have established a rat model mimicking repetitive and intermittent sleep deprivation combined with unpredictable stress in humans. We evaluated anxiety-related behavior and cognitive behavioral parameters and provided neuropathological evidences for cognitive dysfunction in this model. Also, we tested the effects of the mood stabilizers drugs, Li and VPA, in the prevention of these abnormalities.

## Materials and Methods

### Animals

The experiments were performed with 80 male *Wistar* periadolescent rats (weight: 50–60 g) obtained from the Animal House of Universidade Federal do Ceara, Brazil. Animals were housed at a maximum of five per cage in standard polycarbonate cages (42 × 20.5 × 20 cm) and standard environmental conditions (22 ± 1°C; humidity 60 ± 5%; 12-h light/dark cycle with lights on at 7:00 am) with access to food (Laboratory RodentDiet—LabDiet^®^) and water *ad libitum*. All behavioral procedures were conducted between ZT2 (8 am) and ZT5 (11 am) by raters blinded to the experimental groups. The methods were carried out in accordance with the NIH Guide for the Care and Use of Laboratory Animals (32) and with the approval of the local ethical committee of Universidade Federal do Ceara. All efforts were made to minimize animal suffering and to reduce the number of animals used.

### Behavioral Analyses of High and Low Exploratory Rats

A psychogenic selection was employed to study the bases of temperament in rodents, dividing them into extremes of baseline exploratory activity based on the screening for locomotor response to novelty, as previously described (9, 33). The study started with 80 periadolescent animals on the 30^th^ postnatal day (PN30). These animals were selected according to their performance in the open field apparatus for 10 min. Specifically, periadolescent rats were classified into three groups based on their vertical exploratory activity values (33) into high exploratory (HE) (75^th^ percentile), medium exploratory (ME) (50^th^ percentile), and low exploratory (LE) (25^th^ percentile) (**Supplementary Material—Table S1**). Animals were tested again in the same apparatus in adult life (PN60), and those who maintained the same exploratory profile were randomly divided in groups for behavioral and neurochemical evaluations.

### Drugs

Valproic acid (VPA, Tocris, USA) and lithium carbonate (Li; Sigma-Aldrich Corp., St Louis, USA) were used. The drugs were prepared freshly within 1–2 h of dosing. All other chemicals used were of analytical grade.

### Experimental Protocol

**Figure 1:**
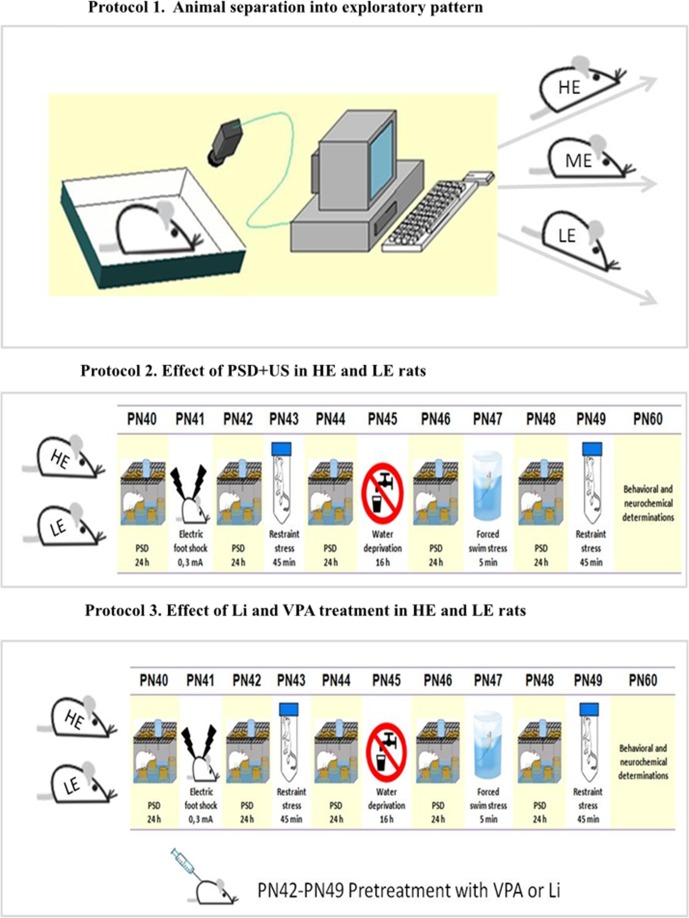
Schematic representation of the experimental design. Protocol 1: On PN (postnatal day) 30, rats were separated according to exploratory activity in HE (high exploratory), ME (medium exploratory), and LE (low exploratory). Protocol 2: From PN40–49, the animals were exposed to daily alternated sections of paradoxal sleep deprivation (PSD) and unpredictable stress (US), day 40—PSD; day 41—foot shock; day 42—PSD; day 43—restraint stress; day 44—PSD; day 45—water deprivation; day 46—PSD; day 47—forced swim stress; day 48—PSD; and day 49—restraint stress or left undisturbed (controls). From PN42–49, some animals were administered valproate (VPA) or lithium (Li), as preventive strategies. The behavioral and neurochemical analysis were performed in brains from animals euthanized on PN60.

The experiments were divided into three protocols as depicted in **Figure 1**. In protocol 1, the animals were selected by exploratory profile, namely, HE, ME, and LE, as previously described. In protocol 2, the animals grouped as HE and LE were divided into four experimental groups (*n* = 8/group), being exposed or not to paradoxical sleep deprivation (PSD) and unpredictable stressors (US): group 1—HE-control (not-stressed), group 2—HE-PSD+US, group 3—LE-control (not-stressed), group 4—LE-PSD+US. In protocol 3, in order to evaluate the effects of Li and VPA in the prevention of behavioral and neurochemical alterations induced by the exposure of HE- and LE-rats to PSD+US, four further groups were conducted, named group 5—HE-VPA+PSD+US, group 6—HE-Li+PSD+US, group 7—LE-VPA+PSD+US, and group 8—LE-Li+PSD+US.

In the present study, since our interest was in studying behavioral and biochemical consequences of the exposure of animals from extremes of temperament to environmental stressors, ME-control rats were used only for comparisons with HE- and LE-control rats. For this reason, we did not conduct a group of ME-rats exposed to PSD+US. For the environmental stress (ES) induction, the animals were subjected to a 10-day protocol with a five alternative day exposure to PSD combined with 5 days of US (as represented in **Figure 1**). Some groups exposed to PSD+US underwent a prevention treatment with the intraperitoneal administration of the mood-stabilizing drugs Li (47.5 mg/kg) or VPA (200 mg/kg). The administration of Li or VPA took place 30 min before the second section of PSD (PN42) and continued until the last day of stress exposure (PN49). The doses of VPA (35) and Li (36, 37) were based on previous studies.

The method of PSD used here was an adaptation of the multiple platform method developed for rats (38). Groups of five to six animals were placed in water tanks (41 cm × 34 cm × 16.5 cm) containing 12 platforms (3 cm in diameter) each, surrounded by water up to 1 cm beneath the surface, for 24 h beginning on PN40 and implemented on alternate days. The present study adopted 24 h of PSD since previous studies showed that it was able to induce hyperlocomotion (taken as a mania-like behavior) (39). In this method, the animals could move inside the tank, jumping from one platform to the other. Control animals were maintained in their home cages in the same room. Food and water were made available through a grid placed on top of the water tank.

On alternate days, beginning on PN41, the animals were submitted to the following sessions of US (40), as detailed in the **Supplementary Material**: day 41—foot shock, day 43—restraint stress, day 45—water deprivation, day 47—forced swim stress, and day 49—restraint stress. From PN50–59, the animals were left undisturbed, and on PN60, the behavioral tests were conducted.

### Behavioral Tests

#### Plus Maze Test

This test was classically designed to evaluate anxiety-like behavior (41). The apparatus has a total dimension of 1,000 (W) × 1,000 (D) × 1,000 (H) mm with two perpendicular open arms and two perpendicular closed arms, 100 (W) × 450 (D) mm arm’s length, with a 650 (H) mm elevation from the floor. The open and closed arms are connected by a central platform. The platform and the lateral walls of the closed arms are made of transparent acrylic. The floor is made of black acrylic. After the respective treatment, each animal was placed at the center of the elevated plus maze with its nose in the direction of one of the closed arms and was observed for 5 min according to the following parameters: number of entries into open and closed arms and the amount of time spent by in open and closed arms of the maze. These data were used to calculate: % of open entries (open entries/total entries × 100), % of time in open arms (time spent in open arms/total time × 100) and % time in the closed arms (time spent in closed arms/total time × 100).

#### Y-Maze Test

This test was used to evaluate working memory (42). Each rat could freely move through the maze during 8 min. The series of arm entries was recorded visually. Alternations were defined as entries in all three arms on consecutive occasions. The percentage of alternation was calculated as total of alternations/(total arm entries − 2) (43).

#### Novel Object Recognition Test (NOR)

This test is widely used to assess the ability to memorize and recognize new and already known objects, being related in rodents to declarative memory (44). On the first day, before any procedure (habituation), animals were placed in the center of the open field apparatus and left for 5 min with no object. After 24 h (training session), animals were placed back in the box with two identical objects (A1 and A2, double Lego^®^ toys) placed in the middle of the box and were left for 5 min for the exploration of the new environment. The time spent exploring each object was recorded for further analysis through the recognition rate. On the same day, 1.5 h after the training session, rats were tested for short-term memory. In this procedure, animals were placed back into the box, with two similar objects (A1 and B1) in color and size, but of different shapes. Again, the animals were left to explore the objects for 5 min and the exploration time of each object was registered. To analyze the results, we used the recognition index, which is calculated as follows: T(B1)  − T (A1)/TE, where T(B1) is the time spent by the animal in exploring the new object (B1), T(A1) is the time spent exploring the familiar object (A1), and TE is the total operating time (the sum of animal exploration time in the old and new objects) (45). The test was used to evaluate short-term memory.

#### Neurochemical and Plasma Determinations

Immediately after the memory tests, the rats were euthanized and the hippocampus was dissected and homogenized for neurochemical determinations.

#### Determination of Reduced Glutathione (GSH) Levels

The levels of GSH were evaluated to estimate endogenous defenses against oxidative stress. The method was based on Ellman’s reagent (DTNB) reaction with free thiol groups (46). The reaction was read in the absorbance of 412 nm, and the product was expressed as ng of GSH/mg wet tissue.

#### Measurement of Lipid Peroxidation

Lipid peroxide formation was analyzed by measuring the thiobarbituric acid reacting substances (TBARS) in brain homogenates (47), as an index of reactive oxygen species (ROS) production. Lipid peroxidation was assessed by the absorbance at 532 nm and expressed as μmol of malonaldehyde (MDA)/mg of tissue.

#### Nitrite Levels

This method is based on the use of the Griess reagent, which detects the presence of nitrite in the sample through a diazotization reaction by formation of a chromophore pink color. The reagent is prepared using equal parts of 5% phosphoric acid, 0.1% N-1-naphthalenediamine (NEED), 1% sulfanilamide in 5% phosphoric acid, and distilled water. The assay was performed by the addition of 100 μl of the supernatant of the homogenate (diluted 10% in potassium phosphate buffer) to 100 μl of Griess reagent. The standard curve was obtained through serial dilutions (100, 50, 25, 12.5, 6.25, 3.12, 1.56 μM) of nitrite. The entire assay was performed in a 96-well plate, and absorbance readings were taken in the 560-nm range. Nitrite product is expressed as μmol of nitrite/mg of tissue (48).

#### Immunoassay for Interleukin (IL)-1**β**, IL-4, and IL-6

Brain tissues were homogenized in 8 volumes of PBS buffer with protease inhibitor (EMD Biosciences) and centrifuged (10,000 rpm, 5 min) for the collection of the supernatant. The concentration of cytokines (IL-1β, IL-4, and IL-6) in 50 ml samples was determined by Enzyme-Linked Immunosorbent Assay (ELISA) immunoenzymatic assays (R&D Systems, Minneapolis, MN, USA), according to the manufacturer’s protocol, and expressed in pg/g tissue in hippocampal samples and in pg/ml in plasma samples.

#### Determination of Uric Acid

In the case of uric acid, the determination was based on the Cobas method. In this enzymatic colorimetric assay, uricase cleaves uric acid to form allantoin and hydrogen peroxide. In the presence of peroxidase, 4-aminophenazone is oxidized by hydrogen peroxide to a quinone-diimine dye. The red color intensity of the quinone-diimine formed is directly proportional to the uric acid concentration and is determined by measuring the increase in absorbance. The results of plasma levels of uric levels are expressed in mg/dl.

### RNA Isolation for Gene Expression Analyses

Total RNA was isolated from hippocampal samples using the SV Total RNA Isolation System from Promega (Madison, WI, USA). RNA was quantified by NanoDrop (Thermo Fisher Scientific), and RNA quality was determined by examining the 260/280 ratio > 1.8. A total of 1 µg RNA was then reverse transcribed using a high-capacity cDNA reverse transcription Kit (Applied Biosystems), according to the manufacturer’s protocol. Messenger RNA (mRNA) expression was analyzed by quantitative PCR (qPCR) according to the manufacturer’s instructions (Applied Biosystems, USA). The sequences of primers and ID numbers are listed in **Table 1**. Target gene expression was calculated relative to a stably expressed reference glyceraldehyde 3-phosphate dehydrogenase (*Gapdh* gene). A total of 50 ng cDNA was added to a mix containing the gene expression assay [1 µl indoleamine 2, dioxygenase 1 (*Ido1*), tryptophan 2,3-dioxigenase (*Tdo2*), inducible nitric oxide synthase (*Inos*), or *Gapdh*, Power SYBR Green PCR master mix (10 µl), and RNA-free water (5 µl) to a final volume of 20 µl]. After the reaction components were mixed by inverting the tube several times, the tube was briefly centrifuged. Then, 20 µl of PCR reaction mix was transferred to each well of a 96-well plate, which was loaded into the instrument, sealed, and centrifuged. The Light cycler 96 (Roche) thermo cycler parameters were 50°C for 2 min and 95°C for 10 min, followed by 40 cycles of 95°C for 15 s and 60°C for 60 s. All fold changes were calculated by the ΔΔC_t_ method (49).

**Table 1:** PCR amplification primers.

Gene name	GenBank accession no.	Forward primer 5′-3′	Reverse primer 5′-3′	Fragment size
iNOS	NM_012611.3	AGGCCACCTCGGATATCTCT	TGGGTCCTCTGGTCAAACTC	81
IDO1	NM_02397.1	CATCAAGACCCGAAAGCACT	GGTGTCTGGATCCACGAAGT	108
TOD2	NM_022403.2	GGGGGATCCTCAGGCTATTA	GGGAACCAGGTACGATGAGA	96

### Data Analysis

The division of the animals, according to the exploratory activity, into HE, ME, and LE groups was based on frequency distribution. For the comparisons between HE-, ME-, and LE-control animals (results of Protocol 1), we used one-way ANOVA followed by Dunnett’s multiple comparisons test, considering ME values as control column. Data obtained in Protocol 2 were analyzed by two-way ANOVA followed by Tukey *post hoc* test considering as factors “exploratory activity” (HE and LE) and “stress exposure” (no-stress and PSD+US). The effects of Li or VPA treatments in HE or LE rats (protocol 3) were analyzed by one-way ANOVA with Tukey as *post hoc* test. Data are presented as means ± standard error (S.E.M.). GraphPad Prism version 6.0 was used for data analyses and construction of graphs. The alpha level was set at 0.05.

## Results

### HE- and LE-Rats Present Distinct Anxiety-Related Behavior That is Prevented by the Administration of the Mood-Stabilizing Drugs VPA and Li

**Figure 2:**
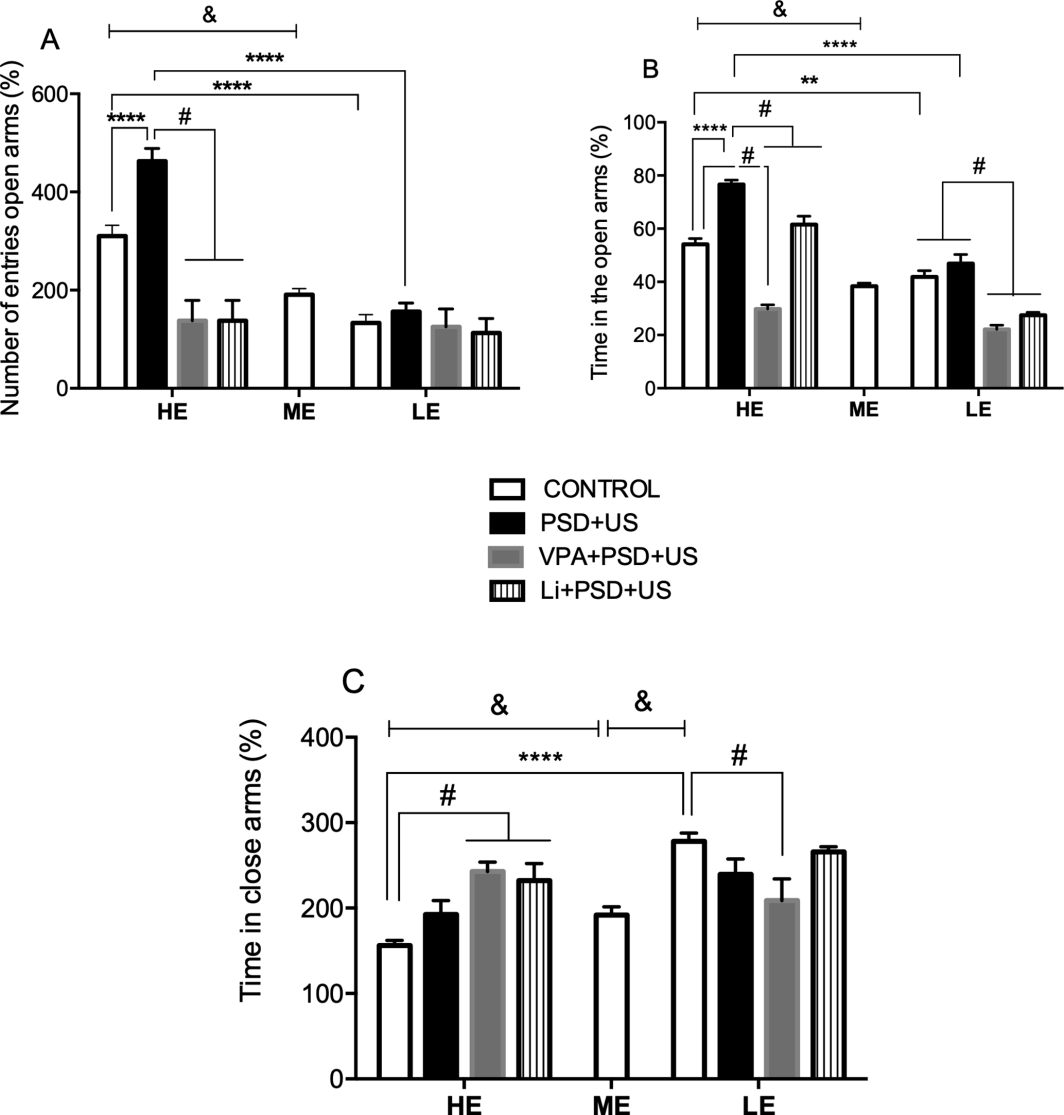
Effects of the exposure of HE- and LE-rats to PSD+US in the plus maze test for the evaluation of anxiety-related behavior. The parameters measured were as follows: % of the number of entries in open arms **(A)**, % of the time in open arms **(B)**, and % of time in closed arms **(C)**. Animals were separated by exploratory pattern in HE (high exploratory), ME (medium exploratory), and LE (low exploratory). HE and LE rats, respectively, mimic the human temperaments hyperthymic and depressive. During periadolescence, PNs 40–49, HE- and LE-rats were exposed to daily alternated sections of paradoxal sleep deprivation (PSD) and unpredictable stress (US) or left undisturbed (controls). The preventive strategies, VPA or Li, were administered during PNs 42–49. Plus maze test was performed on PN60. Bars represent means ± SEM of eight animals/group. ^&^ *P* < 0.05 for comparisons between HE-, ME-, and LE- control rats according to one-way ANOVA followed by Dunnett *post hoc* test; **P < 0.01, ****P < 0.0001 for two-way ANOVA comparisons with Tukey as *post hoc* test using “exploratory activity” and “stress exposure” as factors; ^#^ *P* < 0.05 for comparisons between groups pretreated with Li- and VPA- in relation to PSD+US and controls according to one-way ANOVA followed by Tukey *post hoc* test. HE, high exploratory; ME, medium exploratory; LE, low exploratory; PN, postnatal day; PSD, paradoxal sleep deprivation; US, unpredictable stress; VPA, valproate; Li, lithium.

In the analysis of the % of open arms entries in the plus maze test (**Figure 2A**), we observed that HE-control rats presented increased levels of this parameter relative to ME-control rats {*P* < 0.0001; one-way ANOVA [F (2, 26) = 25.89, *P* < 0.0001]}. The exposure of HE-rats to PSD+US induced a 1.5-fold increase in the % of open entries in relation to HE-controls {*P* < 0.0001; two-way ANOVA “exploratory activity” vs. “stress exposure” interaction [F (1, 31) = 9.412, *P* = 0.0044]}. The administration of VPA or Li significantly prevented PSD+US-induced increase in the % of open entries in HE-rats {HE-VPA+PSD+US vs. HE-PSD+US, *P* = 0.0075; HE-Li+PSD+US vs. HE-PSD+US, *P* = 0.0075; one-way ANOVA [F (2, 29) = 8.077, *P* = 0.0027]}.

In the analysis of the % of time in open arms (**Figure 2B**), HE-control rats presented a significant increase in this parameter in relation to ME-control rats {*P* < 0.0001; one-way ANOVA [F(2, 26) = 18.12, *P* < 0.0001]}. In the analysis of the interaction between “exploratory activity” and “stress exposure,” we observed that HE-control rats presented a significant increase in the % of time in open arms in relation to LE-control rats (*P* = 0.0038) and also that the exposure of HE-rats to PSD+US caused a significant 1.4-fold increase in the % of time spent in the open arms relative to HE-control rats {HE-PSD+US vs. HE-control, *P* < 0.0001; two-way ANOVA interaction: [F(1, 31) = 12.59, *P* = 0.0016]}. The administration of VPA or Li to HE-PSD+US rats prevented from the alteration induced by PSD+US {*P* < 0.0001; one-way ANOVA [F(3, 29) = 52.17, *P* < 0.0001]}. No significant changes were observed by the exposure of LE-rats to PSD+US in relation to LE-control. Despite this, the administration of VPA or Li decreased the % of time in the open arms when compared to LE-control and to LE-PSD+US {*P* < 0.0001; one-way ANOVA [F(3, 29) = 23.65, *P* < 0.0001]}.

In the evaluation of the % of time spent in the closed arms (**Figure 2C**), we observed that HE-control rats presented a significant decrease in this parameter when compared to ME-control (*P* < 0.05), whereas LE-control presented increased levels relative to ME-rats (*P* < 0.0001). We also observed that HE-control presented lower % of time in closed arms in relation to LE-control {*P* < 0.0001; two-way ANOVA interaction [F (1, 31) = 8.788, *P* = 0.0059]}. The pretreatment with Li (*P* = 0.0027) or VPA (*P* = 0.0006) significantly increased the % of time in the closed arms of HE-PSD+US rats in relation to HE-control rats [one-way ANOVA F (3, 29) = 8.352, *P* = 0.0004], whereas LE-PSD+US rats pretreated with VPA presented decreased % time in closed arms when compared to LE-control {*P* = 0.0214; one-way ANOVA [F (3, 29) = 3.587, *P* = 0.0265]}.

### The Exposure to PSD+US Impairs Memory Performance in HE- and LE-Rats That Is Distinctly Prevented by the Mood-Stabilizing Drugs

**Figure 3:**
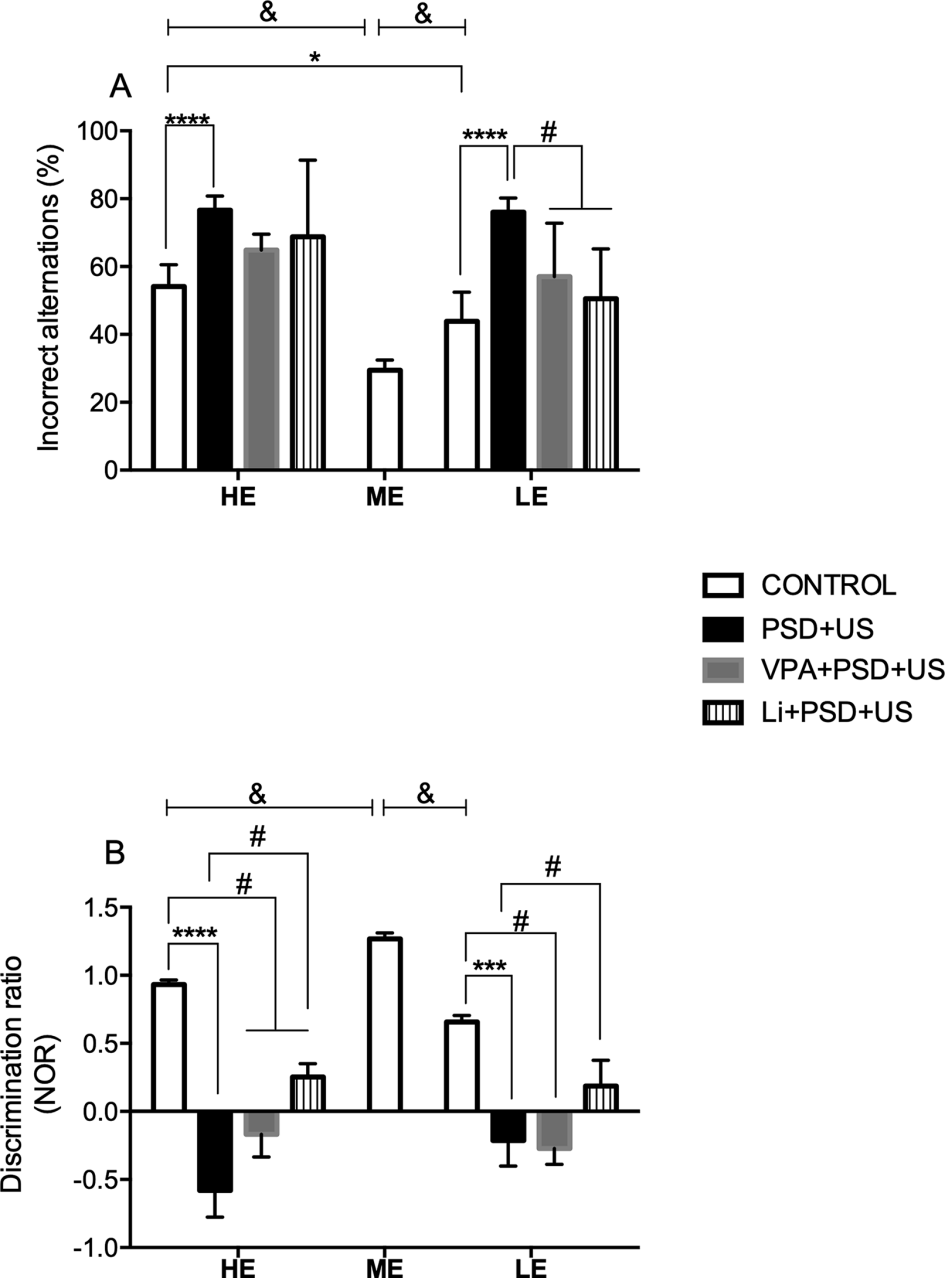
Effects of the exposure of HE and LE rats to PSD+US in memory alterations evaluated by the % of incorrect alternations in the Y maze task **(A)** or discrimination ratio in the novel object recognition test (NOR) **(B)**. Animals were separated by exploratory pattern in HE (high exploratory), ME (medium exploratory), and LE (low exploratory). HE and LE rats, respectively, mimic the human temperaments hyperthymic and depressive. During periadolescence, PNs 40–49, HE- and LE-rats were exposed to daily alternated sections of paradoxal sleep deprivation (PSD) and unpredictable stress (US) or left undisturbed (controls). The preventive strategies, VPA or Li, were administered during PNs 42–49. Y maze task and novel object recognition test (NOR) were performed on PN60 for the evaluation of declarative memory and working memory, respectively. Bars represent means ± SEM of eight animals/group. ^&^ *P* < 0.05 for comparisons between HE-, ME-, and LE- control rats according to one-way ANOVA followed by Dunnett *post hoc* test; *P < 0.05, ***P < 0.001, ****P < 0.0001 for two-way ANOVA comparisons with Tukey as *post hoc* test using “exploratory activity” and “stress exposure” as factors; ^#^ *P* < 0.05 for comparisons between groups pretreated with Li- and VPA- in relation to PSD+US and controls according to one-way ANOVA followed by Tukey *post hoc* test. HE, high exploratory; ME, medium exploratory; LE, low exploratory; PN, postnatal day; PSD, paradoxal sleep deprivation; US, unpredictable stress; VPA, valproate; Li, lithium.

In the analysis of working memory (**Figure 3A**), we observed that both HE- (*P* < 0.0001) and LE-control (*P* = 0.0070) presented increased % of incorrect alternations when compared to ME-control {one-way ANOVA [F (2, 22) = 26.82, *P* < 0.0001]}. Two-way ANOVA indicated a significant “exploratory activity” vs. “stress exposure” interaction [F (1, 28) = 4.472, *P* = 0.0435]. *Post hoc* analysis showed that the percentage of incorrect alternations in HE-PSD+US and LE-PSD+US was significantly increased in relation to controls matched by exploratory activity (*P* < 0.0001). Additionally, HE-control rats presented increased % of incorrect alternations in relation to LE-control (*P* = 0.0104). Pretreatment with Li or VPA did not prevent working memory deficits in HE-rats, while prevented working memory deficits in LE-rats {LE-VPA+PSD+US vs. LE-PSD+US, *P* = 0.0129; LE-Li+PSD+US vs. LE-PSD+US, *P* = 0.0016; one-way ANOVA [F (3, 27) = 12.02, *P* < 0.0001]}.

As depicted in **Figure 3B**, we observed that both HE- and LE-control presented significant lower discrimination index relative to ME-control {*P* < 0.0001; one-way ANOVA [F (2, 17) = 52.27, *P* < 0.0001])}. Both HE-PSD+US (*P* < 0.0001) and LE-PSD+US (*P* = 0.0009) presented marked decreases in discrimination indexes when compared to controls matched by exploratory activity {two-way ANOVA interaction [F (1, 20) = 5.236, *P* = 0.0331]}. Pretreatment of HE-PSD+US rats with Li (HE-Li+PSD+US vs. HE-control, *P* = 0.0076) or VPA (HE-VPA+PSD+US vs. HE-control, *P* < 0.0001) maintained the significant decrease in discrimination ratio in relation to HE-control rats, while pretreatment of HE-PSD+US rats with Li increased the discrimination index in relation to HE-PSD+US group (*P* = 0.0019). Again, LE-VPA+PSD+US group presented decreased discrimination index when compared to LE-control (*P* = 0.0021) being this result akin to LE-PSD+US levels, whereas LE-Li+PSD+US group presented significant increased levels of discrimination index relative to LE-PSD+US group (*P* = 0.0216).

### The Exposure of HE- and LE-Rats to PSD+US Increases Hippocampal Levels of GSH, Lipid Peroxidation, and Nitrite, Which Are Distinctly Altered by the Administration of Mood-Stabilizing Drugs

**Figure 4:**
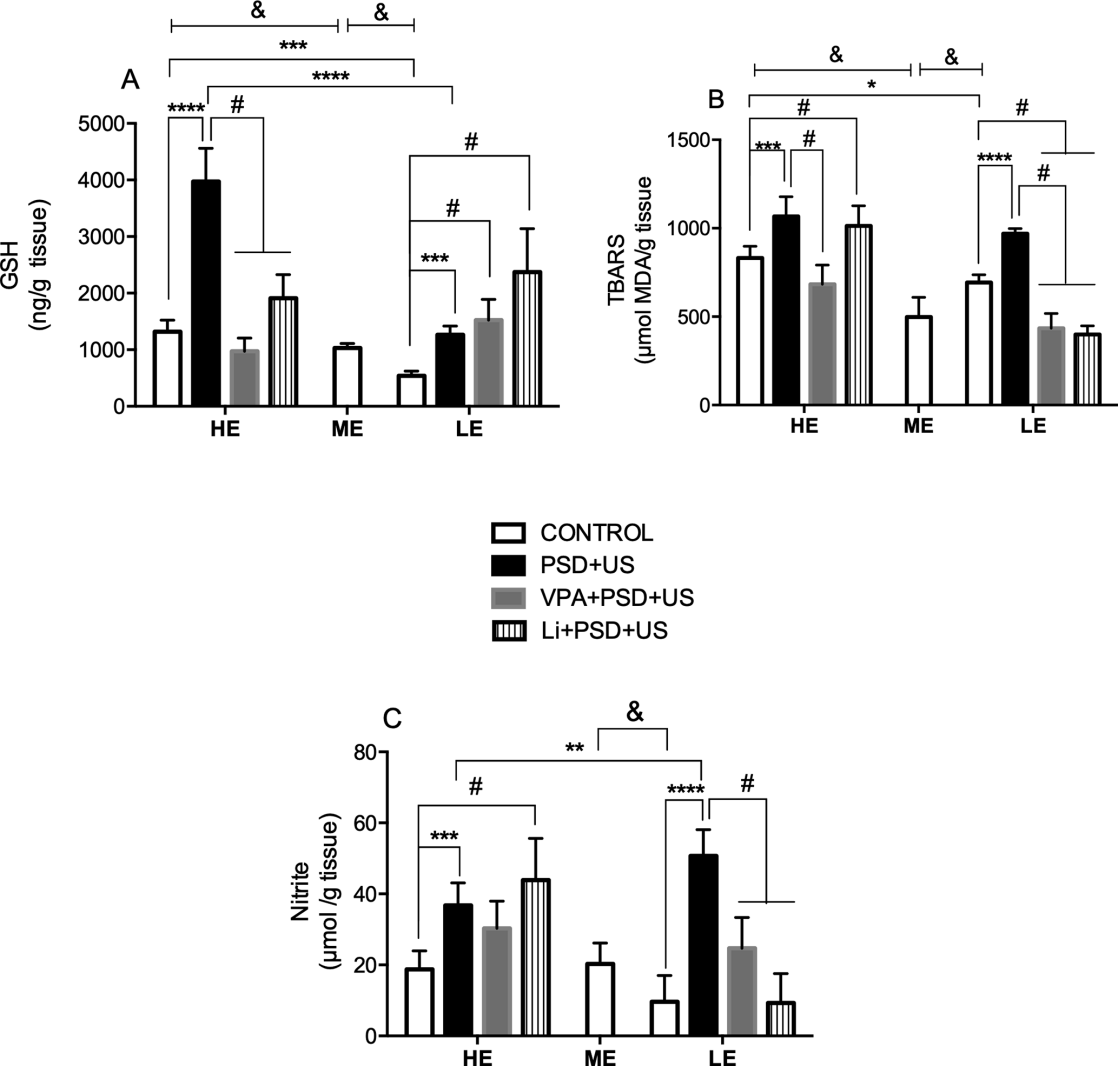
Effects of the exposure of HE and LE rats to PSD+US in oxidative parameters, namely, GSH **(A)**, thiobarbituric acid reacting substances (TBARS) (for lipid peroxidation) **(B)**, and nitrite **(C)**. Animals were separated by exploratory pattern in HE (high exploratory), ME (medium exploratory), and LE (low exploratory). HE- and LE-rats, respectively, mimic the human temperaments hyperthymic and depressive. During periadolescence, PNs 40–49, HE- and LE-rats were exposed to daily alternated sections of paradoxal sleep deprivation (PSD) and unpredictable stress (US) or left undisturbed (controls). The preventive strategies, VPA or Li, were administered during PNs 42–49. The hippocampi were dissected on PN60. Bars represent means ± SEM of six to eight animals/group. ^&^ *P* < 0.05 for comparisons between HE-, ME-, and LE-control rats according to one-way ANOVA followed by Dunnett *post hoc* test; *P < 0.05, ***P < 0.001, ****P < 0.0001 for two-way ANOVA comparisons with Tukey as *post hoc* test using “exploratory activity” and “stress exposure” as factors; ^#^ *P* < 0.05 for comparisons between groups pretreated with Li- and VPA- in relation to PSD+US and controls according to one-way ANOVA followed by Tukey *post hoc* test. HE, high exploratory; ME, medium exploratory; LE, low exploratory; PN, postnatal day; PSD, paradoxal sleep deprivation; US, unpredictable stress; VPA, valproate; Li, lithium.

In the evaluation of hippocampal levels of GSH (**Figure 4A**), we observed that HE-control presented increased levels of this antioxidant defense relative to ME-control (*P* = 0.0008). On the other hand, decreased levels of GSH were observed in LE-control when compared to ME-control {*P* < 0.0001; one-way ANOVA [F (2, 19) = 79.41, *P* < 0.0001]}. There was a significant interaction between “exploratory activity” and “stress exposure” in GSH levels {two-way ANOVA [F (1, 22) = 71.41, *P* < 0.0001]}. In this regard, HE-control presented significant increased hippocampal levels of GSH when compared to LE-control (*P* = 0.0003). We detected a 3- and 2.3-fold increase in GSH levels, respectively, in HE-PSD+US and LE-PSD+US rats in relation to their controls (*P* < 0.0001). Furthermore, the exposure of HE-rats to PSD+US caused a marked increase in GSH levels that was significant in relation to LE-PSD+US (*P* < 0.0001). The administration of VPA or Li to HE-PSD+US rats prevented the alterations in GSH levels (*P* < 0.0001). On the contrary, the administration of these drugs to LE-PSD+US induced a further increase in the levels of GSH relative to LE-controls (*P* < 0.05).

Regarding lipid peroxidation (**Figure 4B**), we detected decreased levels of MDA in the hippocampus of ME-control in relation to LE- (*P* = 0.0004) and HE-control {*P* < 0.0001; one-way ANOVA [F (2, 18) = 31.45, *P* < 0.0001]}. HE-control presented increased hippocampal MDA levels when compared to LE-control (*P* = 0.0358). Both HE-PSD+US (*P* = 0.0008) and LE-PSD+US (*P* < 0.0001) presented increased levels of lipid peroxidation in relation to their respective controls {two-way ANOVA, significant main effect of “exploratory activity” [F (1, 23) = 9.819, *P* = 0.0047] and “stress exposure” [F (1, 23) = 54.86, *P* < 0.0001]}. VPA pretreatment significantly prevented PSD+US alterations in HE-rats, while both VPA and Li prevented the alterations induced by PSD+US in LE-rats, and also decreased the levels of MDA in relation to LE-control (*P* < 0.05).

Nitrite levels (**Figure 4C**) were decreased in LE-control relative to ME ones {*P* = 0.0085; one-way ANOVA [F (2, 18) = 5.985, *P* = 0.0102]}. Both HE- and LE-rats when exposed to PSD+US presented increased hippocampal levels of nitrite when compared to their respective controls (HE-PSD+US vs. HE-control, *P* = 0.0002; LE-PSD+US vs. LE-control, *P* < 0.0001). Furthermore, the levels of nitrite in LE-PSD+US rats were significantly increased when compared to HE-PSD+US {*P* = 0.0030; two-way ANOVA interaction [F (1, 23) = 20.70, *P* = 0.0001]}. The administration of both mood-stabilizing drugs prevented nitrite increase in LE-PSD+US rats (LE-VPA+PSD+US vs. LE-PSD+US, *P* < 0.01; LE-Li+PSD+US vs. LE-PSD+US, *P* < 0.0001), while Li increased the levels of nitrite in HE-PSD+US rats (HE-Li+PSD+US vs. HE-PSD+US, *P* < 0.01).

### Exposure to PSD+US Increases Cytokine Levels in the Hippocampus and Plasma as Well as Uric Acid in the Plasma of HE-Rats

**Figure 5:**
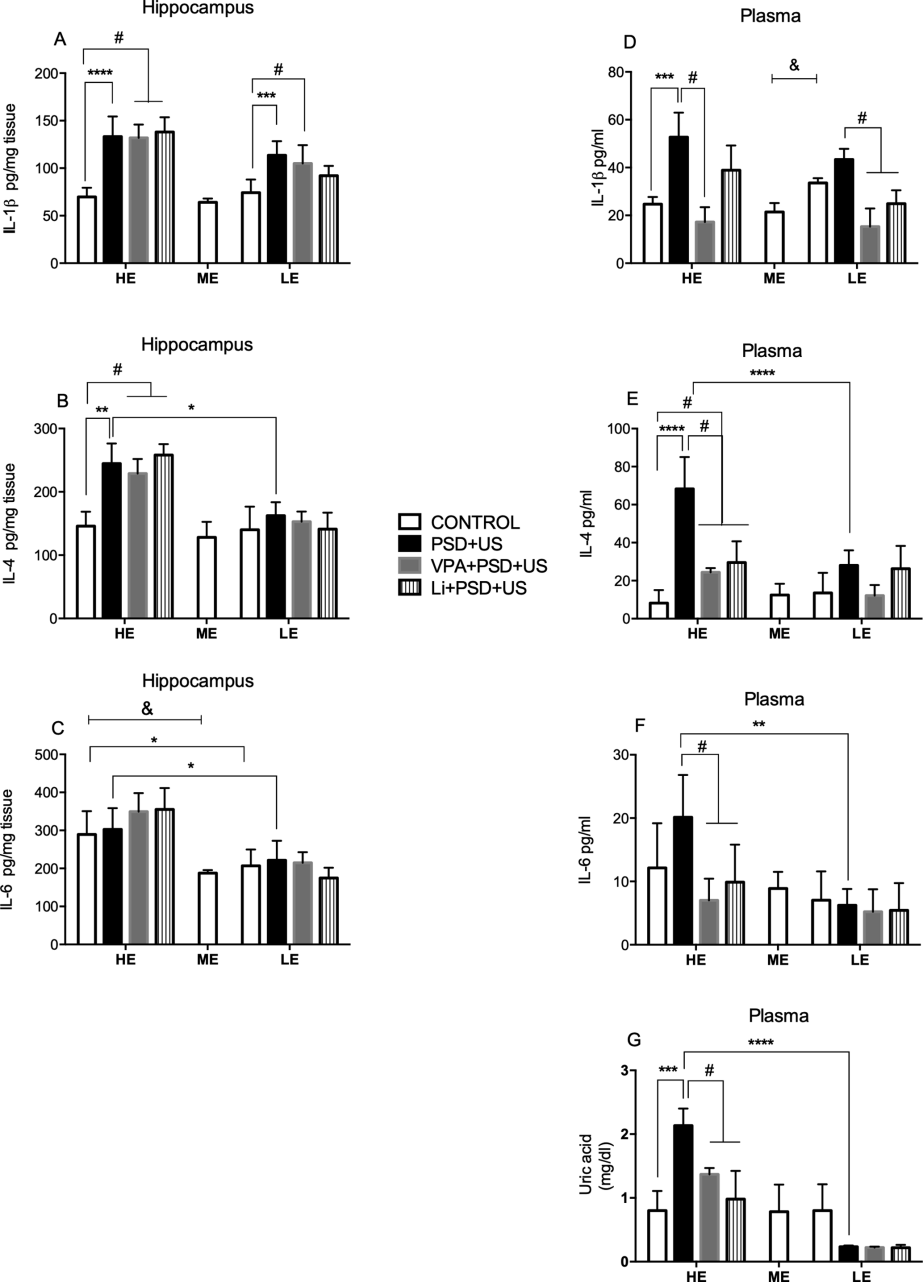
Effects of the exposure of HE and LE rats to PSD+US in hippocampal IL-1β **(A)**, IL-4 **(B)**, IL-6 **(C)** and plasma IL-1β **(D)**, IL-4 **(E)**, IL-6 **(F)**, uric acid **(G)** inflammatory parameters. Animals were separated by exploratory pattern in HE (high exploratory), ME (medium exploratory), and LE (low exploratory). HE- and LE-rats, respectively, mimic the human temperaments hyperthymic and depressive. During periadolescence, PNs 40–49, HE- and LE-rats were exposed to daily alternated sections of paradoxal sleep deprivation (PSD) and unpredictable stress (US) or left undisturbed (controls). The preventive strategies, VPA or Li, were administered during PNs 42–49. The hippocampi and blood samples were collected on PN60. Bars represent means ± SEM of six to eight animals/group. ^&^ *P* < 0.05 for comparisons between HE-, ME-, and LE-control rats according to one-way ANOVA followed by Dunnett *post hoc* test; *P < 0.05, **P < 0.01, ***P < 0.001, ****P < 0.0001 for two-way ANOVA comparisons with Tukey as *post hoc* test using “exploratory activity” and “stress exposure” as factors; ^#^ *P* < 0.05 for comparisons between groups pretreated with Li- and VPA- in relation to PSD+US and controls according to one-way ANOVA followed by Tukey *post hoc* test. HE, high exploratory; ME, medium exploratory; LE, low exploratory; PN, postnatal day; PSD, paradoxal sleep deprivation; US, unpredictable stress; VPA, valproate; Li, lithium.

We found a significant increase in the hippocampal levels of IL-1β (**Figure 5A**) in both HE- and LE-rats exposed to PSD+US in relation to their respective control {HE-PSD+US vs. HE-control, *P* < 0.0001; LE-PSD+US vs. LE-control, *P* = 0.0002; two-way ANOVA significant main effect of “stress exposure” [F (1, 33) = 69.63, *P* < 0.0001]. The administration of the mood-stabilizing drugs did not prevent the alterations in this parameter. Regarding hippocampal IL-4 (**Figure 5B**), we found increased levels of this cytokine in HE-PSD+US rats relative to HE-control (*P* = 0.0012), being this increase also significant in relation to LE-PSD+US {*P* = 0.0103; two-way ANOVA interaction [F (1, 25) = 4.713, *P* = 0.0396]}. Again, the mood-stabilizing drugs could not prevent IL-4 changes in HE-PSD+US rats. Hippocampal IL-6 levels (**Figure 5C**) were increased in HE-control in relation to ME-control {*P* = 0.0002, one-way ANOVA [F (2, 23) = 12.81, *P* = 0.0002]}. We observed that IL-6 levels were higher in HE-control relative to LE-control (*P* = 0.0119) and in HE-PSD+US in relation to LE-PSD+US {*P* = 0.0207; two-way ANOVA significant main effect of “exploratory activity” [F (1, 30) = 20.57, *P* < 0.0001]}.

Plasma levels of IL-1β (**Figure 5D**) were increased in LE-control when compared to ME-control {*P* = 0.0010; one-way ANOVA [F (2, 15) = 13.96, *P* = 0.0017]}. We observed a marked increase in IL-1β levels in HE-PSD+US animals in relation to HE-control {*P* = 0.0001; two-way interaction [F (1, 13) = 8.077, *P* = 0.0139]}. Pretreatment with VPA prevented the alterations in IL-1β induced by the exposure of HE-rats to PSD+US (*P* < 0.05). On the other hand, the administration of the mood-stabilizing drugs decreased the levels of this cytokine in LE-rats (*P* < 0.01). IL-4 levels (**Figure 5E**) were significantly increased in HE-rats exposed to PSD+US in relation to HE-control (*P* < 0.0001). This increase observed in HE-PSD+US rats was also significant in comparison with LE-PSD+US rats {*P* < 0.0001; two-way ANOVA interaction [F (1, 27) = 9.736, *P* = 0.0043]}. The administration of both mood-stabilizing drugs decreased IL-4 plasma levels in relation to HE-PSD+US animals (*P* < 0.001). In the analysis of plasma IL-6 levels (**Figure 5F**), we observed that HE-PSD+US rats presented increased levels of this cytokine in relation to LE-PSD+US group {*P* = 0.0081; two-way ANOVA significant main effect of “exploratory activity” [F (1, 22) = 13.92, *P* = 0.0012]}. We also observed a decrease in IL-6 levels by the administration of VPA and Li to HE-PSD+US rats (*P* < 0.05).

Uric acid plasma levels (**Figure 5G**) were two-fold higher in HE-PSD+US rats in relation to HE-control (*P* = 0.0004), being this increase also significant in relation to LE-PSD+US {*P* < 0.0001; two-way ANOVA interaction [F (1, 22) = 22.13, *P* = 0.0001]}. Pretreatment with VPA (*P* = 0.0045) and Li (*P* = 0.0002) prevented the increase in uric acid levels in HE-rats. No significant alterations were observed in LE-rats.

### PSD+US Alters the mRNA Expression of the Enzymes iNOS, IDO1, and TDO2 in the Hippocampus of HE- and LE-Rats

Since we observed that the exploratory activity of rats influences brain and plasma cytokines’ levels, we decided to evaluate the expression of the enzymes involved in tryptophan metabolism, regulated by pro-inflammatory environment (50), and by glucocorticoids, namely, IDO1 and TDO2, respectively. We also evaluated the expression of iNOS, because it is increased in an inflammatory environment, and also based on the five-fold increase in nitrite levels detected in the hippocampus of LE-PSD+US rats.

**Figure 6:**
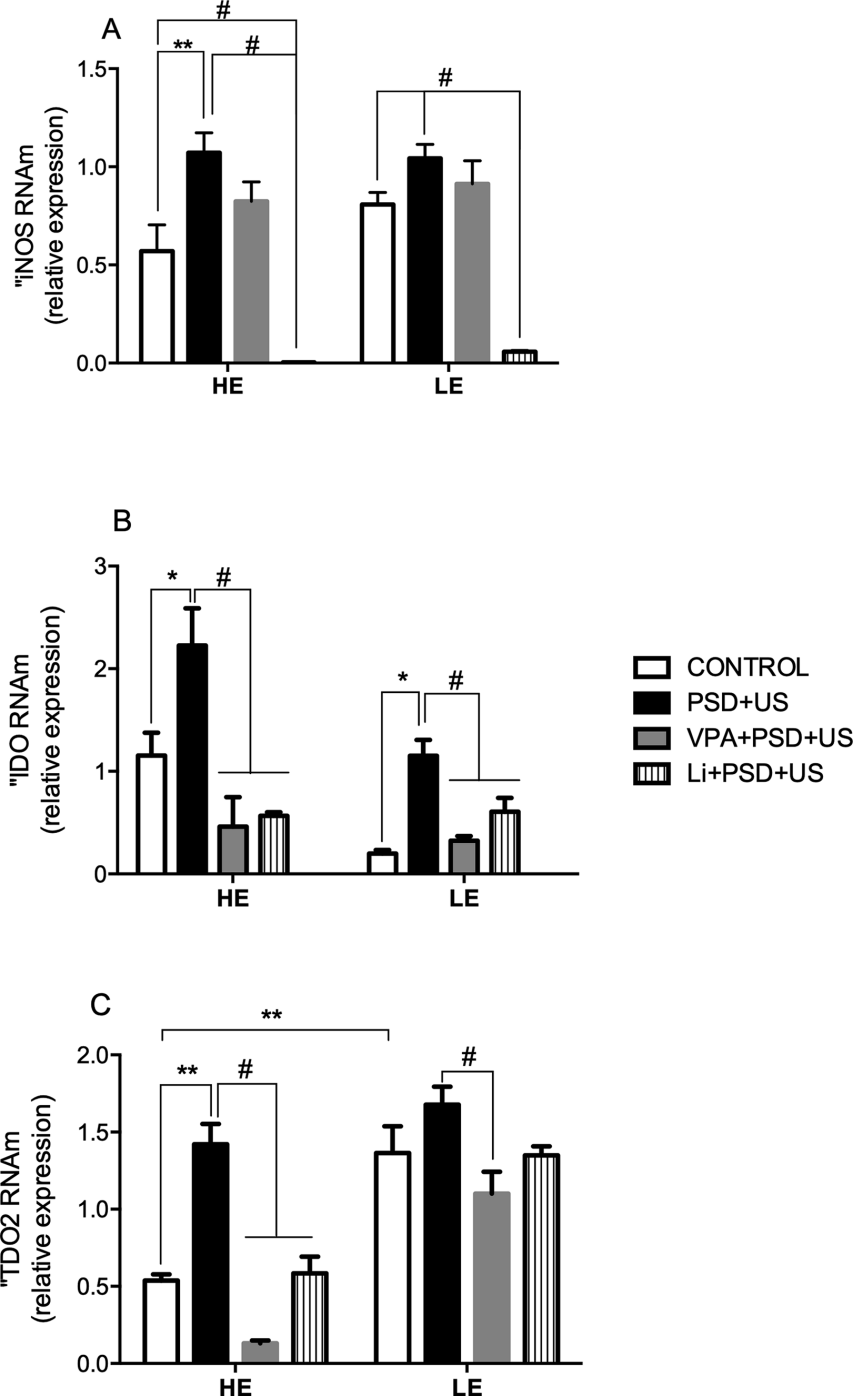
Effects of the exposure of HE and LE rats to PSD+US in iNOS **(A)**, IDO **(B)**, and TDO **(C)** mRNA relative expression. Animals were separated by exploratory pattern in HE (high exploratory), ME (medium exploratory), and LE (low exploratory). HE- and LE-rats, respectively, mimic the human temperaments hyperthymic and depressive. During periadolescence, PNs 40–49, HE- and LE-rats were exposed to daily alternated sections of paradoxal sleep deprivation (PSD) and unpredictable stress (US) or left undisturbed (controls). The preventive strategies, VPA or Li, were administered during PNs 42–49. The hippocampi were dissected on PN60. Bars represent means ± SEM of five animals/group. *P < 0.05, **P < 0.01 for two-way ANOVA comparisons with Tukey as *post hoc* test using “exploratory activity” and “stress exposure” as factors; ^#^ *P* < 0.05 for comparisons between groups pretreated with Li- and VPA- in relation to PSD+US and controls according to one-way ANOVA followed by Tukey *post hoc* test. HE, high exploratory; ME, medium exploratory; LE, low exploratory; PN, postnatal day; PSD, paradoxal sleep deprivation; US, unpredictable stress; VPA, valproate; Li, lithium.

The exposure to environmental stressors significantly increased iNOS mRNA levels in HE-rats when compared to their respective control (HE-PSD+US vs. HE-control, *P* = 0.0078). Pretreatment with Li prevented iNOS increased expression in HE-PSD+US and also reduced its expression to minimum levels (*P* < 0.01). A similar reduction in iNOS expression was observed in the group LE-Li+PSD+US when compared to LE-control (*P* < 0.0001) (**Figure 6A**).

We detected a significant two-fold increase in IDO mRNA levels in both HE-PSD+US (*P* = 0.02) and LE-PSD+US (*P* = 0.0379) when compared to their respective controls. Pretreatment with VPA or Li significantly prevented the alterations in HE-rats (*P* < 0.001) (**Figure 6B**). In relation to TDO2 (**Figure 6C**), HE-PSD+US rats presented an almost three-fold increase in the expression of this enzyme when compared to HE-control, while both VPA and Li prevented this alteration (*P* < 0.001). On the other hand, the expression of TDO2 was higher in LE-controls in relation to HE groups (*P* < 0.01). VPA pretreatment of LE-rats decreased TDO2 expression when compared to PSD+US group (*P* < 0.01).

## Discussion

Variations in temperament are core traits that can be passed to descendants and lead to a greater risk of developing bipolar disorder (51). In line with this evidence, affective temperaments may help predict vulnerability to mood disorders, while exposure to stress triggers mood episodes (52). In the present study, we observed that the exposure of HE-rats (a pattern of exploratory activity in rats that mimics hyperthymic temperament) and LE-rats (a pattern of exploratory activity in rats that mimics depressive temperament) (8) to a combination of two distinct types of stressors, namely, PSD and US, causes distinct alterations in anxiety-related behavior, but similar working memory and declarative memory impairments accompanied by inflammatory and oxidative changes that varies according to the animals’ exploratory profile. In other words, HE-rats exposed to PSD+US presented with more hippocampal pro-inflammatory alterations in relation to LE-rats, as evidenced by increased expression of iNOS and higher plasma levels of IL-1β and uric acid. Furthermore, HE-PSD+US rats presented a marked increase in plasma IL-4 levels. The effects of mood stabilizers appeared to be related to temperament. Indeed, both VPA and Li prevented LE-PSD+US rats from working memory impairment, while the alterations in declarative memory were partially prevented only by Li in both LE- and HE-PSD+US rats. Regarding hippocampal oxidative parameters, both VPA and Li prevented GSH alterations induced by PSD+US in HE-rats, while alterations in lipid peroxidation and nitrite levels were prevented by both drugs only in LE-rats. Plasma alterations of IL-4 and uric acid observed in HE-PSD+US rats were prevented by both drugs, while hippocampal IDO expression induced by PDS+US was prevented by both drugs in animals of both temperaments.

### Evaluation of Anxiety-Related Behavior in HE and LE Rats Exposed to Environmental Stressors

In the present study, we observed increased percentage of open arms entries in the plus maze in HE-rats when compared to ME- and LE-rats, an indicative of anxiolytic behavior. Furthermore, when exposed to PSD+US, these rats presented a marked increase in this parameter that was prevented by both mood-stabilizing drugs, VPA and Li. Notably, this anxiolytic pattern observed in HE-rats may be related in patients to an increased risk-taking behavior that is observed in mood disorders, such as bipolar disorder and replicated in animal models of this disorder (53). On the contrary, LE-rats presented increased tendency to explore closed arms, which was not influenced by the exposure to environmental stressors.

In the last years, some attempts have been made to develop models of mood disorders based on the exposure of animals to environmental stressors. In this regard, the exposure of adult rodents (not selected by exploratory pattern) to chronic US is related to the development of depressive-like symptoms (54, 55), while the exposure to PSD is related to the occurrence of mania-like symptoms (39, 56). Based on this evidence, previous studies have been conducted with the exposure of adult rodents to US or PSD, separately. A recent study of our research group showed that the exposure of adult rats to a combination of PSD and hot air blast (HAB) (57), this last being a stressful stimulus related to the development of depressive-symptoms, can induce behavioral and memory alterations that resemble bipolar disorder endophenotypes (58).

In the present study, we exposed periadolescent animals to a combination of stressors. Adolescence is a developmental period characterized by significant neuronal and adrenocortical maturation (59–61). A previous study evaluating the consequences of one-night sleep deprivation on adolescent neurobehavioral performance found that one night of total sleep deprivation had significant deleterious effects upon neurobehavioral performance and subjective sleepiness, leading to impairment in daytime functioning (62). Indeed, sleep loss is known to trigger mania or depression (63). In adolescents, the acute glucocorticoid response, both in human and in rodents, requires a longer period to return to baseline levels compared to adults and prepubertal youth. Hence, adolescence is a developmental period for programming of adult adrenocortical responses (59, 63–65).

### Contributions of Temperament Combined With Periadolescence Exposure to Environmental Stressors to the Development of Memory Impairment and Hippocampal Oxidative/Inflammatory Alterations

Our study showed that the exposure of periadolescent rats from extremes of temperaments (HE- and LE-rats) to PSD+US (for 10 days) impaired working and declarative memory. We also observed that the increase in the percentage of incorrect alternations and lower discrimination ratio observed in HE- and LE-rats was significant in relation to ME-controls. Taken together, our data suggest that the temperament itself induces working and declarative memory dysfunctions that are exacerbated by repeated and intermittent exposure to PSD+US. Indeed, extensive studies have shown an association between sleep deprivation (66–68) or US (69–72) and cognitive processes in humans and animals (73–77). In line with our findings, various studies have shown sleep deprivation-induced learning and memory impairments in different tests, such as avoidance tasks (78–80), Morris water maze (81, 82), radial maze (73), and the plus-maze discriminative avoidance tasks (83–85). Thus, both acute and chronic forms of sleep deprivation seem to interfere with cognitive functions. In addition, numerous studies have demonstrated that US in laboratory animals produces memory deficits in several behavioral models, such as the Morris water maze (86, 87), Y-maze (86), and elevated plus maze (86, 88).

We also observed that LE-controls presented a lower percentage of incorrect alternations in the Y-maze task when compared to HE-controls. This greater incidence of errors in the Y-maze observed in HE-controls may be related to the higher impulsivity of these rats (89), and also confirmed in our results, since high impulsives are more likely to be involved in risky behavior than low impulsive subjects (90).

Adult patients with obstructive sleep apnea have been shown to present decreased levels of antioxidants and lower performance on neuropsychological tasks (91). A recent meta-analysis revising studies on sleep deprivation and oxidative stress in animals revealed that in rats, 11 out of 13 reports showed oxidative alterations in at least one brain region. In this meta-analysis, one study showed decreased lipid peroxidation, whereas 10 studies showed an increase in oxidative stress (92).

Some molecular mechanisms may underlie the memory impairments induced by sleep deprivation and US. Several studies showed that the cognitive decline triggered by these environmental contingencies could be attributed to a hippocampal oxidative imbalance or a decrease in synaptic plasticity *via* the reduction of brain-derived neurotrophic factor (BDNF) levels (93) after sleep deprivation (94–97) or US (98–100). Thus, as an attempt to explore the mechanisms involved in the memory impairments observed by the exposure to both stressors (PSD+US), in the present study, we measured oxidative/immune-inflammatory parameters in order to better understand the impact of hippocampal changes triggered by the exposure of periadolescent animals with extreme temperaments to these two environmental stressors.

In our results, we observed that temperament alters oxidative homeostasis. Indeed, HE- and LE-controls when compared to ME-controls presented increased levels of hippocampal lipid peroxidation, while LE-controls showed decreased nitrite and GSH levels. On the other hand, both HE- and LE-rats presented increased lipid peroxidation and nitrite levels when exposed to PSD+US. The levels of GSH, the main antioxidant defense (37), were increased in both HE- and LE-PSD+US US groups, but in HE-rats, PSD+US-induced increase was greater when compared to LE-rats. A previous study searching for the time course of memory impairments and oxidative changes in animals exposed to carbon monoxide showed that in the initial period, these animals presented increments in lipid peroxidation and also increased levels of GSH during the course of memory impairment (93). We hypothesize that this increase in hippocampal GSH levels may be a compensatory mechanism to prevent excessive brain damage. This hypothesis is built on the fact that HE-rats present increased baseline brain levels of dopamine (101), which is at least partially responsible for the increased locomotor activity of these animals, whereas dopamine metabolism generates reactive oxygen species that may lead to brain damage (102). In line with this increase in dopamine metabolism in HE-rats, we observed here that HE-control presents increased levels of GSH when compared to ME- (1.3-fold) and LE-controls (2.4-fold) as well as presented higher levels of lipid peroxidation (1.7-fold in relation to ME and 1.4-fold in relation to LE).

Since the neurobiology of mood disorders involves both oxidative and inflammatory alterations (103, 104), we decided to study hippocampal and plasma levels of inflammatory markers. We observed that the hippocampal levels of IL-1β were increased in both HE- and LE-rats exposed to PSD+US, but this hippocampal change did not completely correlate with the alterations observed in the plasma levels of this cytokine. Indeed, since the increase in IL-1β levels was greater in the hippocampus of HE-PSD+US rats compared HE-control (almost two-fold change), and LE-PSD+US presented a 1.5-fold increase in IL-1β compared to LE-control, we believe that the increase in plasma levels of HE-rats and not LE-rats may reflect the difference in the magnitude of the brain IL-1β changes observed between HE- and LE-rats.

The extremes of temperament also influenced IL-4 brain and plasma levels. We observed that only HE-PSD+US rats presented increased hippocampal (1.6-fold increase) and plasma levels of IL-4 (8.5-fold increase). IL-4 is a cytokine related to memory function (105). While normal levels of IL-4 may have neuroprotective effects, high levels of this cytokine are observed in extreme situations, such as traumatic brain injury (106). Hence, IL-4 also presents pro-inflammatory activity (105). IL-4 is a T helper type (Th) 2 cytokine. Recently, Th2 response has been implicated in memory impairments based on the positive correlations between aging and memory deficits with eotaxin-1/CCL11 levels. To date, eotaxin-1/CCL11 is considered an endogenous cognitive deteriorating chemokine whose levels are increased in neurodegenerative disorders and schizophrenia (107). IL-4 induces the release of eotaxin-1/CCL11 from eosinophils and can cross the unaltered blood-brain barrier (108). Eotaxin-1/CCL11 may shift the immune response toward Th2 and promote microglia migration and activation with subsequent production of reactive oxygen species, potentiating glutamate-induced neuronal death (109).

Reinforcing the increase in plasma pro-inflammatory alterations in HE-PSD+US rats, we observed a marked increase in uric acid. Indeed, a previous study searching for deleterious effect of uric acid in memory revealed that a diet based on high-uric acid triggers the expression of proinflammatory cytokines, activates the Toll-like receptor 4 (TLR4)/nuclear factor (NF)-κB pathway, and increases gliosis in the hippocampus of *Wistar* rats (110). Furthermore, high uric acid levels have been associated with hyperthymic and irritable temperament scores, whereas low uric acid levels associate to depressive temperament scores. This finding has led to the assumption that uric acid levels may be a biological marker for the differentiation of unipolar and bipolar disorder (111).

Still in line with the immune-inflammatory changes, we observed that while both HE- and LE-rats exposed to PSD+US presented increased hippocampal expression of the enzyme IDO, only HE-PSD+US rats presented increased hippocampal expression of iNOS and TDO2. This result is in line with the hippocampal levels of cytokines (112), since we observed increased levels of IL-1β in both HE- and LE-PSD+US rats and increased IL-4 only in HE-PSD+US rats. On the other hand, TDO2 activity is associated with Th2 response. Similarly, NO modulates Th1/Th2 balance (113). Since increased expression of iNOS leads to an overproduction of NO, this mechanism of increased Th2 response may also be influenced by iNOS. As previously discussed, only HE-PSD+US rats presented increased hippocampal and plasma levels of IL-4. Hence, the results obtained in the present study suggest an association between memory impairment observed in HE-PSD+US rats and a Th2 mechanism. This needs to be further explored in future studies.

### Effect of Mood-Stabilizing Drugs in the Prevention of Memory Alterations and Oxidative/Immune-Inflammatory Mechanisms in HE- and LE-Rats Exposed to PSD+US

The results of the present study showed that Li treatment partially prevented PSD+US-induced impairment in declarative memory in HE- and LE-rats, while both VPA and Li prevented working memory deficits only in LE-PSD+US group. We also measured the levels of cytokines and ROS to investigate possible mechanisms in VPA and Li’s preventive effects on hippocampus-dependent learning and memory impairment in HE and LE rats. As a result, we found that VPA decreased plasmatic levels of IL-1β and IL-6 in HE rats. There was also a decrease of IL-1β levels in LE animals by both Li and VPA. Moreover, VPA was able to reduce the levels of lipid peroxidation in HE and LE rats. Interestingly, plasma levels of the cytokines IL-1β and IL-4 as well as of uric acid were predictive of the effects of the mood-stabilizing drugs only in HE-rats.

Taken together, these results show the role of VPA and Li in neuronal functions, neuroprotection, learning, and memory.

The present study has some limitations since we did not test the effects of each stressor separately. The reason for this decision was to follow the principle of the 3Rs, since there is a great amount of literature on US and PSD single exposure. Another limitation was that the protocol was conducted only in males and not in female rats. We also did not evaluate microglia alterations, nor the levels of eotaxin-1/CCL11 and corticosterone, which seems to be important parameter for determination in future studies.

In conclusion, our findings contribute to a limited body of literature investigating the cognitive consequences of sleep deprivation alternated with unpredictable stress in rats separated by temperament, as well as its response to mood stabilizers. Our results are congruent with the current knowledge that exposure to environmental stressors and temperamental susceptibility triggers mood disorder, and extend current literature, suggesting that a Th1/Th2 imbalance may contribute to neuroinflammatory and oxidative changes in HE and LE rats. In this context, the study of affective temperament, such as its genetic bases, phenotypic alterations, and influence in the treatment of mood disorders, becomes an instrument for personalized psychiatry.

## Data Availability

All datasets generated for this study are included in the manuscript and the supplementary files.

## Ethics Statement

The methods were carried out in accordance with the NIH Guide for the Care and Use of Laboratory Animals (12) and with the approval of the local ethical committee of Universidade Federal do Ceara. All efforts were made to minimize animal suffering and to reduce the number of animals used.

## Author Contributions

CL, FS, AC, AQ, and AO treated the animals and performed the behavioral evaluations and neurochemical analyses. CL also helped in the study design. CL, FS, SV, and DFL performed the statistical analyses. GF, JQ, and MF contributed to the study design and to the first draft. DM designed the study, constructed the graphics, and wrote the final version.

## Conflict of Interest Statement

The authors declare that the research was conducted in the absence of any commercial or financial relationships that could be construed as a potential conflict of interest.
