# Supplementary material for: High Exploratory Phenotype Rats Exposed to Environmental Stressors Present Memory Deficits Accompanied by Immune-Inflammatory/Oxidative Alterations: Relevance to the Relationship Between Temperament and Mood Disorders
Source: Front Psychiatry. 2019 Aug 5;10:547. doi: 10.3389/fpsyt.2019.00547 (PMC6689823; doi:10.3389/fpsyt.2019.00547)
Supplement: Supplementary file 1 [file DataSheet_1.docx]

Supplementary Material

**PERIADOLESCENSE UNPREDICTABLE STRESS (US) EXPOSURE**

This procedure was adapted from elsewhere, see(9). US was induced using a variable sub-chronic stress protocol, which included five distinct stressors applied on alternate days beginning on PN41.The five distinct stressors are described in detail below and were applied in the following order: 1. Electric foot shock (day 41); 2. Restraint stress (day 43); 3. Water deprivation (day 45); 4. Forced swimming stress (day 47); 5. Restraint stress (day 49) (9).

*Exposure to electric foot shock (PN41):* We used a passive avoidance apparatus (Brazil model EP-104-MC ). Each animal was first placed into the chamber for an initial period of 3 min, after which it received 3 mild electric foot shocks (0.5 mA) separated by intervals of 3 min. The electric foot shocks lasting 1 s. The session ended with an additional 3 min period, in which no shocks were delivered.

*Exposure to restraint stress (PN43):* Each animal was kept for 45 min in transparent plastic tube (diameter: 6 cm, length: 13 cm). The restrainer tubes contained drilled holes (2 mm in diameters) so as to facilitate oxygen supply. The tubes were tapped on a table which was placed in testing room. The animals were immediately returned to their home cages at the end of the restraint stress procedure.

*Exposure to water deprivation (PN45):* Animals were kept in their home cages, and the water bottles were removed at 16h00 on PND44 and added again the next day at 08h00. Animals had free access to food during the entire water deprivation period.

*Exposure to forced swim stress (PN47):* The apparatus used for the forced swim stress was made of a circular white fiberglass tank (diameter: 50 cm, 18 cm in diameter) filled with water (temperature: 18 °C, depth: 30 cm). The water tank was placed in a brightly lit testing room. Each animal underwent two session of forced swimming, each lasting 5 min. The two-swimming sessions were separated by a 5-min interval, during which the animals were kept in a waiting box containing sawdust embedding. The animals were dried with a towel and immediately brought back to their home cages after the second swimming session.

*Exposure to restraint stress (PN49):* Restraint stress was repeated.

Table S1- Minimum, maximum, mean, standard deviation, 25^th^ , 50^th^ and 75^th^ percentile of the temperamental division of animals.

| **Minimum** | **25^th^ Percentile** | **50^th^**  **Percentile** | **75^th^ Percentile** | **Maximum** | **Mean** | **Standard deviation** |
| --- | --- | --- | --- | --- | --- | --- |
| 3,000 | 32,00 | 46,00 | 67,25 | 101,0 | 48,59 | 22,91 |
